# High‐Density Electroanatomical Mapping of Alternating Short‐ and Long‐RP Orthodromic Tachycardia With Identical Atrial Breakthrough

**DOI:** 10.1002/joa3.70440

**Published:** 2026-08-03

**Authors:** Alejandro Carta‐Bergaz, Melanie Fernández‐Caso, Esteban González‐Torrecilla, Ángel Arenal

**Affiliations:** ^1^ Department of Cardiology Hospital General Universitario Gregorio Marañón Madrid Spain; ^2^ Facultad de Medicina, Instituto de Investigación Sanitaria Gregorio Marañón and CIBERCV Universidad Complutense de Madrid Madrid Spain

**Keywords:** accessory pathway, electroanatomical mapping, longitudinal dissociation, orthodromic reciprocating tachycardia, supraventricular tachycardia

## Abstract

High‐density electroanatomical mapping during orthodromic tachycardia with alternating short‐ and long‐VA conduction patterns demonstrated an identical earliest atrial breakthrough. Although this supports a common atrial insertion, it cannot distinguish functional longitudinal dissociation within a single accessory pathway from closely adjacent ventricular pathway components converging into a common atrial insertion.

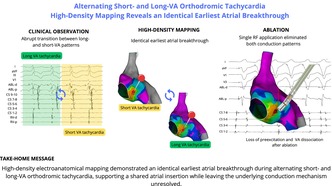

A 44‐year‐old man presented with recurrent palpitations and documented narrow QRS supraventricular tachycardia. Baseline ECG showed intermittent preexcitation through a posteroseptal accessory pathway (Figure 1A).

**FIGURE 1:**
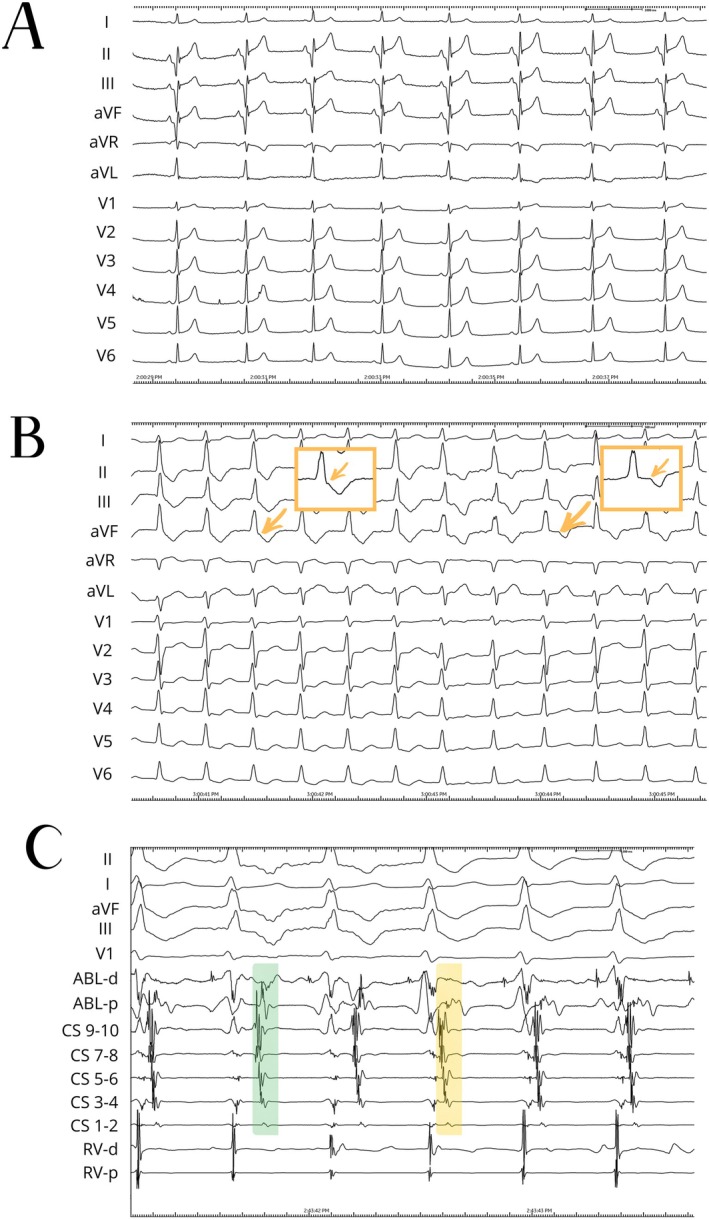
Orthodromic AVRT with dual retrograde conduction. (A) Baseline 12‐lead ECG showing preexcitation consistent with a posteroseptal accessory pathway. (B) Orthodromic tachycardia with spontaneous changes in the RP interval; retrograde P waves are indicated by yellow arrows. (C) Intracardiac recordings during tachycardia demonstrating abrupt ventriculoatrial (VA) interval shifts with preservation of the retrograde atrial activation sequence. Despite marked VA shortening, tachycardia cycle length changed only minimally because of reciprocal AH prolongation.

During an electrophysiological study, the patient's tachycardia, with a cycle length of 375–378 ms, was reproducibly induced with atrial extrastimulus. Abrupt spontaneous transitions between discrete short‐ and long‐VA patterns (122 and 174 ms, respectively, measured from surface V to the earliest atrial electrogram in the proximal coronary sinus) were observed despite an apparently identical retrograde atrial activation sequence in the His bundle and coronary sinus recordings (Figure 1B,C). To clarify the mechanism, high‐density electroanatomical mapping was performed during both conduction patterns.

At baseline, the AH and HV intervals measured 72 ms and 17 ms, respectively. Incremental atrial pacing resulted in progressive preexcitation up to a pacing cycle length of 300 ms, at which point antegrade block in the accessory pathway occurred. Prolonged periods of intermittent preexcitation were observed, frequently initiated by premature beats arising near the coronary sinus. Atrial stimulation reproducibly induced typical AV nodal echo beats, consistent with dual AV nodal physiology. During ventricular pacing, the retrograde atrial activation sequence remained unchanged despite repetitive abrupt short to long ventriculoatrial (VA) jumps at rates of 370 ms (Figure 2A).

**FIGURE 2:**
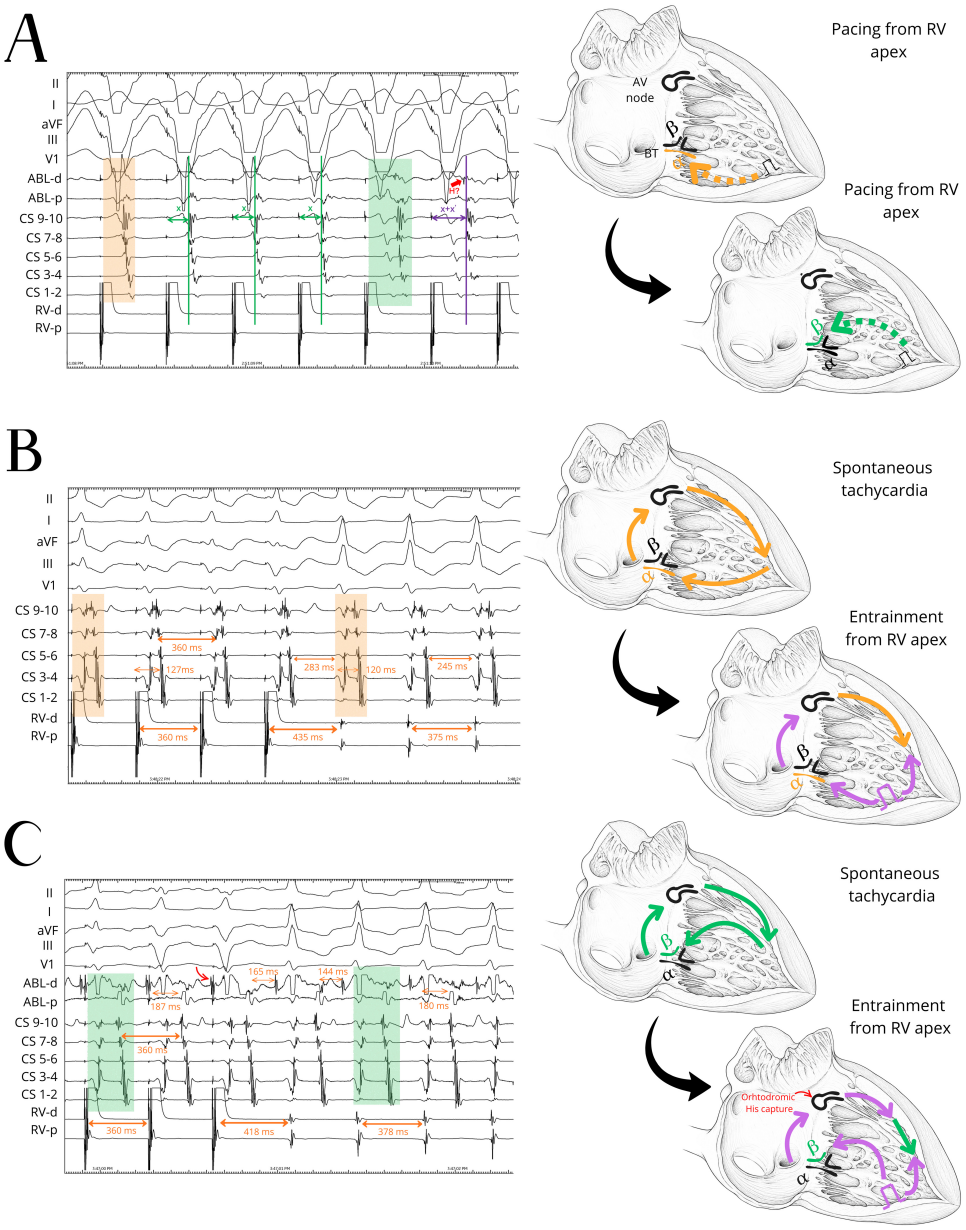
Retrograde VA jump during orthodromic tachycardia with preserved atrial activation sequence. (A) Transition during ventricular burst stimulation at 370 ms from short VA conduction (retrograde limb labeled α) to long VA conduction (retrograde limb labeled β), without change in atrial activation sequence. Jump from short to long VA is abrupt, without progressive prolongation (*x* = 121 ms with short VA; *x* + *x*′ = 172 with long VA). The electrogram marked by the red arrow may represent a retrograde His potential. However, it occurs simultaneously with the earliest atrial activation, making exclusive retrograde AV nodal conduction unlikely. (B) Ventricular entrainment of orthodromic tachycardia with short VA conduction (α pathway), showing a post‐pacing interval (PPI) of 435 ms, tachycardia cycle length (TCL) of 375 ms, and SA–VA difference of 7 ms (127–120 ms); minimal change in postentrainment AV interval (283–245 ms). (C) Ventricular entrainment of orthodromic tachycardia with long VA conduction (β pathway), showing a PPI of 418 ms, TCL of 378 ms, and SA–VA difference of 7 ms (187–180 ms); minimal change in postentrainment AH interval (165–144 ms). The red label shows His orthodromic capture reinforcing orthodromic tachycardia diagnosis. Like in panel (B) there is evident atrial reset during the transition zone (fused surface QRS). N.B.: Intermittent displacement of the coronary sinus catheter occurred, explaining subtle variations in the apparent atrial activation sequence across recordings in (B) and (C). However, whenever catheter position remained stable, the retrograde atrial activation sequence was unchanged as shown in (A). Minor changes in local VA during ventricular entrainment and tachycardia may be explained by an oblique course of the more rapidly conducting retrograde pathway, whereby changes in the ventricular activation wavefront alter the local activation timing.

The differential diagnosis of abrupt short‐ and long‐RP transitions during supraventricular tachycardia includes atypical atrioventricular nodal reentrant tachycardia, atrial tachycardia, orthodromic tachycardia using distinct accessory pathways, or variable retrograde conduction over a single accessory pathway. In the present case, marked VA prolongation resulted in only minimal tachycardia cycle length changes (375–378 ms) because of reciprocal AH shortening (Figure 1). Entrainment from the right ventricular apex during both short‐ and long‐VA tachycardias yielded a V‐A‐V response with post‐pacing intervals compatible with accessory pathway participation (a corrected PPI–TCL of 22 and 19 ms during both the short‐ and long‐VA tachycardias, respectively) (Figure 2B,C). The minimal difference between stimulus–atrial and ventriculoatrial intervals (7 ms for both short and long VA interval tachycardia) further supported orthodromic tachycardia using an accessory pathway (Figure 2B,C). Atrial reset during the transition zone (Figure 2B,C) also demonstrated the presence of an accessory bypass tract. The V‐A‐V response during entrainment, together with the entrainment measurements described above, excluded atrial tachycardia. Likewise, the eccentric retrograde atrial activation sequence and elimination of both tachycardia and preexcitation after posteroseptal ablation made atypical atrioventricular nodal reentry unlikely (Figure 2).

Although distinct accessory pathways could theoretically explain alternating VA intervals, this mechanism would usually be expected to produce differences in retrograde atrial activation sequence or spatially distinct sites of earliest atrial activation. Conventional recordings from the coronary sinus and His bundle catheters may fail to distinguish closely adjacent atrial insertions; therefore, high‐density electroanatomical mapping was performed separately during short‐ and long‐VA conduction patterns using an OCTARAY mapping catheter and the CARTO3 electroanatomical mapping system (Johnson & Johnson MedTech). Separate analysis of electroanatomical maps according to the prevailing VA conduction pattern ultimately demonstrated an identical earliest atrial activation site at the posteroseptal region during both short‐ and long‐VA conduction (Figure 3). During long VA tachycardia a possible Kent potential was identified, suggesting either that markedly slow retrograde conduction within the accessory pathway may functionally mimic a highly oblique pathway course or that the long VA path conducts countercurrent to ventricular activation, thereby masking local VA continuity despite successful ablation at that site (Figure 3) [1]. Radiofrequency application at this site eliminated both retrograde conduction patterns, abolished preexcitation, rendered the tachycardia noninducible, and resulted in VA dissociation during slow ventricular pacing.

**FIGURE 3:**
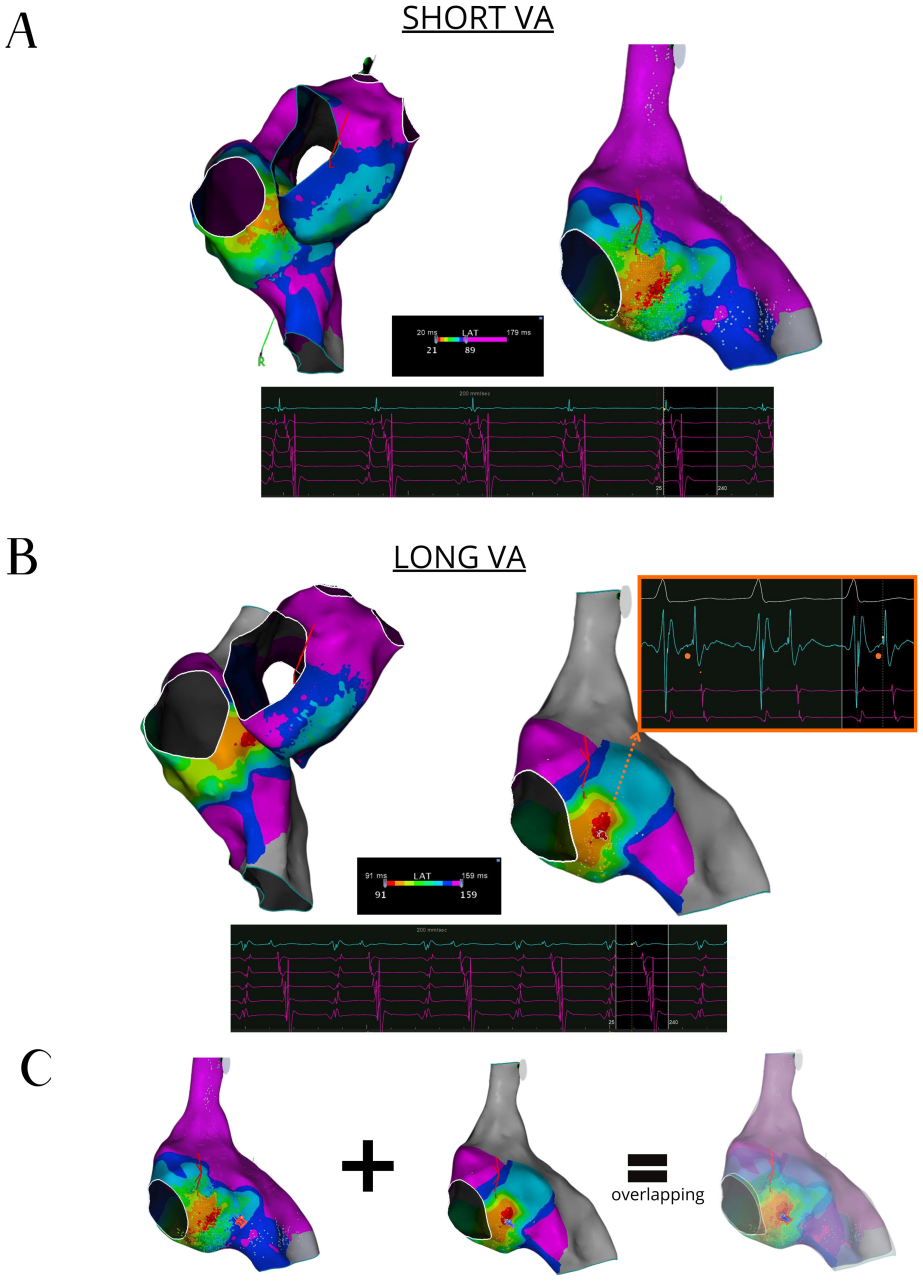
High‐density mapping of short‐ and long‐VA orthodromic tachycardia demonstrating a common site of earliest atrial activation. (A) Electroanatomical atrial activation map using the OCTARAY catheter (2–2–2–2–2 mm spacing) and CARTO3 mapping system during short VA orthodromic tachycardia. (B) Electroanatomical atrial activation map during long VA orthodromic tachycardia. The site of earliest atrial activation (red tag) demonstrated a discrete potential suggestive of an accessory pathway potential (orange dot) and colocalized with the site of earliest atrial activation identified during the long‐VA tachycardia. (C) Superposition of electroanatomical maps in (A) and (B) to facilitate visual comparison of the sites of earliest atrial activation (red tag for the short VA map; blue tag for the VA map).

The mechanism responsible for the abrupt transitions between short‐ and long‐VA conduction patterns cannot be determined with certainty from the available data. Notably, although spontaneous transitions occurred in both directions during tachycardia, during baseline ventricular pacing, conduction more frequently shifted toward the long‐VA pattern. Although speculative, this observation suggests that the switching behavior may not be entirely random. One possible explanation is functional interaction between the two retrograde conduction modes through concealed conduction and linking. Under this hypothesis, conduction over one mode may produce concealed penetration into the other, transiently preventing its participation and thereby maintaining preferential retrograde conduction through a single route. Abrupt switching could occur when this interaction is interrupted because of differential recovery of excitability or functional block.

The electrophysiological findings were most consistent with dual retrograde conduction over a single accessory pathway. Previous reports have described abrupt changes in VA conduction time without modification of the retrograde atrial activation sequence during orthodromic tachycardia or ventricular pacing, findings that have been interpreted as longitudinal dissociation within an accessory pathway [1]. In the present case, abrupt and reproducible transitions between short‐ and long‐VA conduction patterns occurred both spontaneously and during ventricular pacing, fulfilling several of the criteria proposed for longitudinal dissociation [2]. A recent report described spontaneous conversion between long‐ and short‐RP orthodromic tachycardia compatible with this mechanism; however, electroanatomical mapping characterization was not available [3]. In contrast, separate high‐density activation maps in the present case demonstrated an identical site of earliest atrial activation during both short‐ and long‐VA conduction patterns. This observation provides additional insight into the anatomical substrate underlying this phenomenon. Specifically, the identical site of earliest atrial activation makes the presence of two spatially separate accessory pathways with distinct atrial insertions less likely (provided the separation between two theoretical insertions does not lie below the resolution of the catheter and mapping system) and supports the concept that both retrograde conduction patterns converge into a common atrial breakthrough despite markedly different conduction times.

Nevertheless, distinguishing true longitudinal dissociation of a single accessory pathway from closely adjacent ventricular pathway components converging into a common atrial insertion was not possible in this case. Demonstration of an identical ventricular insertion would be required to further support longitudinal dissociation (e.g., by comparing the stimulus‐atrial interval during both short‐ and long‐VA conduction patterns from multiple right‐ and left‐ventricular pacing sites). Importantly, the proposed concealed conduction/linking mechanism is not specific to any particular anatomical substrate and could theoretically operate both in the setting of true longitudinal dissociation and in the presence of extremely closely adjacent pathway components. Other published similar cases have interpreted the differences in VA intervals to be functional dissociation based on the decremental conduction through the bypass tract (in contrast to the discrete VA jumps in our case) but have found the atrial insertion of the accessory pathway to be close but distinct [4]. To our knowledge, this is the first report demonstrating an apparently identical atrial breakthrough during alternating short‐ and long‐VA orthodromic tachycardia using separate high‐density electroanatomical activation maps [3].

This case illustrates how high‐density electroanatomical mapping may provide mechanistic insights into alternating retrograde conduction patterns that cannot be fully resolved by conventional intracardiac recordings alone. Furthermore, activation maps should be analyzed separately according to the prevailing VA conduction pattern, since minimal tachycardia cycle length variations may otherwise lead to inadvertent merging of distinct activation sequences within electroanatomical mapping systems.

## Funding

The authors have nothing to report.

## Ethics Statement

The authors have nothing to report.

## Consent

Written informed consent was obtained from the patient for publication of this case report.

## Conflicts of Interest

Alejandro Carta‐Bergaz reports speaker honoraria from Medtronic. Ángel Arenal reports consulting fees and speaker honoraria from Medtronic, Johnson & Johnson MedTech, and Boston Scientific. The remaining authors declare no conflicts of interest.

## Data Availability

The authors have nothing to report.
